# Study on GIS Visualization in Evaluation of the Human Living Environment in Shenyang-Dalian Urban Agglomeration

**DOI:** 10.1155/2016/7462832

**Published:** 2016-04-20

**Authors:** Kang Hou, Jieting Zhou, Xuxiang Li, Shengbin Ge

**Affiliations:** School of Human Settlements and Civil Engineering, Xi'an Jiaotong University, Xi'an 710049, China

## Abstract

Analysis of human living environmental quality of Shenyang-Dalian urban agglomerations has important theoretical and practical significance in rapid development region. A lot of investigations have been carried for Shenyang-Dalian urban agglomerations, including 38 counties. Based on the carrying capacity of resources, natural and socioeconomic environmental factors and regional changes of human living environmental evaluation are analyzed with the application of geographic information systems (GIS) software. By using principal component analysis (PCA) model and natural breaks classification (NBC) method, the evaluation results are divided into five categories. The results show that the human living environmental evaluation (HLEE) indexes of Dalian, Shenyang, and Liaoyang are higher than other counties. Among these counties, the human living environmental evaluation (HLEE) indexes of coastal counties are significantly higher than inland counties. The range of the human living environmental evaluation index in most of the study area is at III, IV, and V levels, accounting for 80.01%. Based on these results, it could illustrate the human living environment is in relatively suitable condition in Shenyang-Dalian urban agglomeration.

## 1. Introduction

Human Living environmental is closely related to the natural, human, and socioeconomic factors. Since Doxiadis put forward the concept of “Science of Human Living Environment” [1], human living environment has been an important issue in other subjects [2–5]. Human living environment is influenced by many factors, including terrain and landforms, climate, hydrological conditions, and land use/land cover, which play the leading role in the human living environmental evaluation. Most of the research focused on the limit scale and lacked large-scale regional research [6–9]. They also lacked studies on spatial distribution of regional suitability of the human living environment.

Urban agglomeration refers to a certain area, where there is a strong interaction between urban space arrangement forms and individual cities, and it is the product of certain stage of urban development. Shenyang-Dalian urban agglomeration extends into the Bohai Sea and Yellow Sea, which is the largest estuary in Northeast China. Due to its advantage of opening geographical location, it has a very important position in the Northeast Asian region. In process of regional evaluation, the different indicators system will lead to different evaluation results. Therefore, establishing a reasonable evaluation system of human living environment is essential to protect urban agglomeration environment and rationally plan urban construction and development.

Recently, geographic information system (GIS) has been widely used and has become an important environmental evaluation tool [10]. Meanwhile, evaluation models and TM data will be combined through different angles, which can objectively evaluate the complex large-scale regions [11–13]. In this study, the human living environmental assessment model and human living environmental evaluation (HLEE) index based on GIS technology were developed. Then, this research quantitatively analyzed the spatial distribution of human living environmental evaluation index in this urban agglomeration.

## 2. Study Area and Data

### 2.1. The Situation of Study Area

Shenyang-Dalian urban agglomeration was located in Northeast China, near the Bohai Sea and Yellow Sea. This study area is confined by the latitudes 38.701°N~43.525°N and longitudes 121.022°E~125.78°E. The study area is characterized by climatic diversity, including semihumid climate and humid climate, semihumid climate and semiarid climate. The annual average temperature is 5 to 10°C, and the average annual precipitation is 450 mm to 1200 mm by uneven space distribution.

**Figure 1:**
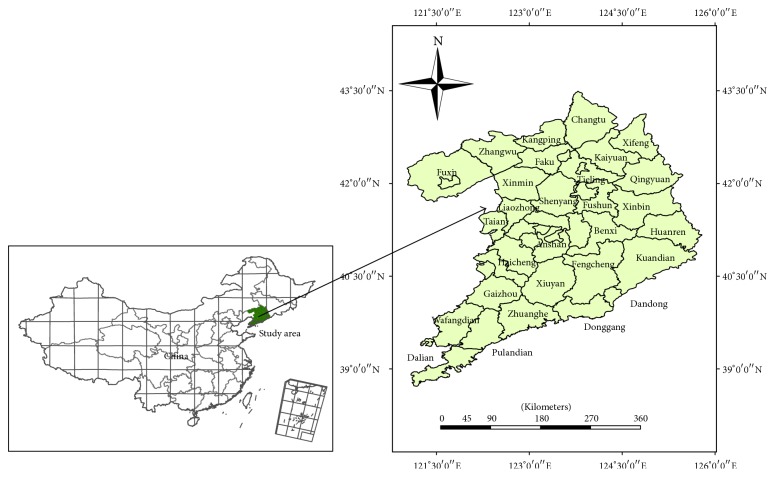
The location of the study area and the main counties.

The area of Shenyang-Dalian urban agglomeration is 104689.84 km^2^. The study area included Shenyang District, Dalian District, Xinmin District, Liaozhong County, Faku County, Kangping County, Anshan District, Haicheng District, Taian County, Xiuyan County, Fushun District, Fushun County, Xinbin District, Qingyuan County, Benxi District, Benxi County, Huanren County, Yingkou District, Gaizhou District, Dashiqiao District, Liaoyang District, Dengta District, Liaoyang County, Tieling District, Kaiyuan District, Tieling County, Xifeng County, Changtu County, Fuxin District, Zhangwu County, Fuxin County, Wafangdian District, Pulandian District, Zhuanghe District, Dandong District, Fengcheng District, Donggang District, and Kuandian County (see Figure 1). Before the 1990s, as the economic development of Northeast China, it is also the country's most important industrial base. Due to the deepening of reform and open policy, Northeast China's economic growth rate gradually fell behind the eastern coastal areas. Development of economy and society in Shenyang-Dalian urban agglomeration can effectively promote the development of the entire Northeast China, and the related research can be a good promotion of socioeconomic development of the region. This is a major initiative to revitalize the Northeast China industrial base.

### 2.2. Data

The main data is Landsat-5 TM remote sensing in 2011, which has a spatial resolution of 30 meters. In addition, the serial numbers of these remote sensing images are 120, 32; 120, 33; 119, 32; 119, 33; and 118, 32. Remote sensing was obtained from the United States Geological Survey; the map projection coordinates were Universal Transverse Mercator. Meanwhile, topography and vegetation coverage data were derived from interpretation of remote sensing data. Meteorological data was obtained from meteorological departments in Shenyang-Dalian urban agglomeration. Socioeconomic data was also acquired from Statistical Yearbooks of these counties in 2011.

## 3. Methods

### 3.1. Influence Factors

In the human living environmental evaluation index system, the choice of evaluation criteria is so important, and it also decides results of the evaluation. These factors should be representative and operable. However, in practice, not all evaluation indexes are so easy to obtain. Therefore, the establishment of the index system should be fully taken into account operability. Human living environmental evaluation system mainly involves natural environmental factors and socioeconomic factors, including population (*C*_1_), land resource (*C*_2_), water resource (*C*_3_), climate (*C*_5_), ecological (*C*_6_), and economy (*C*_8_) [7, 13–15]. The traditional evaluation system can be improved by this assessment system, and it proposes the new evaluation index system, including energy (*C*_4_), public service evaluation (PSE) index (*C*_7_), life and living (*C*_9_), and environment (*C*_10_). So the evaluation system includes three aspects: resource carrying capacity, natural environmental, and socioeconomic factors. Then, these three aspects contain 31 screened factors (see Table 1).

### 3.2. Data Preprocessing

In the multi-index evaluation system, due to the different nature of each index, generally they have different dimensions and magnitude. The original index value is analyzed directly when difference in various indicators is large; it will highlight the role of the higher value of indicators and diminish the role of lower level indicators in a comprehensive analysis system. Therefore, in order to ensure the reliability of the results, the original index data need to be standardized. The original values of these factors were standardized by the following equation:

(1) $$\begin{array}{c} A_{ij}=\frac{B_{ij}-B_{\min,j}}{B_{\max,j}-B_{\min,j}}, \end{array}$$

where *A*_*ij*_ represents the standardized value of factor, *i* and *j* are row and column numbers, *B*_*ij*_ represents the original factor value, and *B*_min,*j*_ and *B*_max,*j*_ represent minimum and maximum value in this column, respectively.

### 3.3. Evaluation Model

Converting 31 factors into a comprehensive evaluation index is a critical step of human living environmental evaluation system. The principal component analysis (PCA) is an objective method of finding this index [16, 17]. Principal component analysis is designed to take advantage of lower-dimensional idea and replace more indicators with a few composite indicators [18]. The number of principal components is less than or equal to the number of original variables. Each of the main components is able to reflect most of the information of the original variables, and then the information does not repeat. The principal components provide information on the most meaningful parameters, which describe the whole data set according to data reduction with minimum loss of original information. This approach will translate several complicating factors into principal component, while the results obtained will be more scientific and effective. In this evaluation system, it contains 31 small evaluation factors. Based on analysis software-SPSS 18.0, principal components will be obtained.

The human living environmental evaluation (HLEE) index is defined as the sum of several weighted principal components as shown below:

(2) $$\begin{array}{c} \text{HLEEI}\left(\;x\;\right)=\overset{7}{\underset{i=1}{\sum }}a_{i}x_{i}, \end{array}$$

where HLEEI(*x*) is human living environmental evaluation index; *a*_*i*_ is principal component weights; *x*_*i*_ is standardized values. And

(3) $$\begin{array}{c} a_{i}=\frac{e_{i}}{\sum_{i=1}^{m}e_{i}}, \end{array}$$

where *e*_*i*_ is the contribution ratio of the *i*th principal component and *i* is the eigenvalue of the *i*th principal component.

### 3.4. Evaluation Index Classification

In order to represent the different human living environmental evaluation levels, the results should be divided into several categories. Based on ArcGIS 9.3 software, the natural breaks classification (NBC) method was applied to the results of evaluation classification in this study. Natural breaks classification is an objective method to analyze the statistical distribution in the attribute space, and this method can be used to identify the classification interval [19, 20]. Similar values are most appropriately grouped, which can maximize the differences in individual classes. In this study, the method of the NBC was used to divide the human living environmental evaluation index into five grades—I, II, III, IV, and V level, and each grade has specific features (see Table 2).

## 4. Result and Discussion

### 4.1. Computing of Human Living Environmental Evaluation Index

According to the cumulative contribution of principal components, the number of components is affirmed to be seven and PCA is accomplished. The corresponding results are shown in Table 3.

The higher the HLEE index value is, the more suitable the human living environment is. Derived from formulae (2) and (3), the human living environmental evaluation index (HLEE index) can be obtained as follows:

(4) HLEEI=0.236F1+0.182F2+0.09F3+0.083F4+0.068F5+0.066F6+0.046F7.

In formula (4), HLEEI is synthetic human living environmental evaluation index and *F*_1_ ~ *F*_7_ are seven principal components from 31 initial spatial variables in 2011.

Human living environmental evaluation index can be obtained from formula (4) in this area. The human living environmental evaluation indexes of Dalian, Shenyang and Liaoyang are higher than other counties. Among these counties, the human living environmental evaluation indexes of coastal counties (including Dandong, Donggang, Pulandian, and Dashiqiao) are significantly higher than those of inland counties (including Fuxin County, Ganzhou, Xinbin, Xifeng County, and Kaiyuan).

### 4.2. Distribution of Human Living Environmental Evaluation Grade

According to the standard (see Table 2), the integrated evaluation indexes are classified to generate corresponding results (see Table 4), as shown in Figure 6. The IV zone with the largest area proportion accounts for 29.99%, the III zone accounts for 28.52%, the V zone accounts for 21.50%, the II zone accounts for 14.65%, and the I zone only accounts for 5.347%. Most of the study area shows that the range of the human living environmental evaluation index is at III, IV, and V levels, which also illustrates the regional human living environment is in relatively good condition.

### 4.3. The Relationship between Main Evaluation Indexes and HLEEI

**Figure 2:**
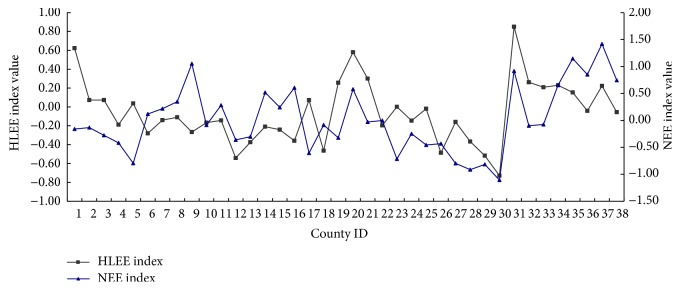
The curve of human living environmental evaluation (HLEE) index and natural environmental evaluation (NEE) index (1: Shenyang, 2: Xinmin, 3: Liaozhong, 4: Faku, 5: Kangping, 6: Anshan, 7: Haicheng, 8: Taian, 9: Xiuyan, 10: Fushun, 11: Fushun County, 12: Xinbin, 13: Qingyuan, 14: Benxi, 15: Benxi County, 16: Huanren, 17: Yingkou, 18: Gaizhou, 19: Dashiqiao, 20: Liaoyang, 21: Dengta, 22: Liaoyang County, 23: Tieling, 24: Kaiyuan, 25: Tieling County, 26: Xifeng, 27: Changtu, 28: Fuxin, 29: Zhangwu, 30: Fuxin County, 31: Dalian, 32: Wafangdian, 33: Pulandian, 34: Zhuanghe, 35: Dandong, 36: Fengcheng, 37: Donggang, and 38: Kuandian).

The difference between natural environmental evaluation (NEE) index and human living environmental evaluation (HLEE) index can be shown in Figure 2. HLEE indexes of Shenyang, Dalian, Liaoyang, and coastal counties are greater than other counties. The curve of HLEE index has relatively large fluctuations, which also reflects that the development of human settlements in Shenyang-Dalian urban agglomeration is uneven. The counties, where NEE indexes are higher, relatively have the larger HLEE indexes, such as Dalian, Liaoyang, Dandong, and Donggang, but the Shenyang, Xiuyan, and Huanren Counties are abnormal. Although the NEE index of Shenyang is lower than others, it is the capital city of Liaoning Province, which has better socioeconomic conditions to make up for weaknesses on the natural environment. On the contrary, the backwardness of socioeconomic conditions in Xiuyan and Huanren leads to the lower HLEE index. The NEE indexes of these old industrial counties are far better than coastal cities, and this difference is mainly caused by the climatic and ecological factors.

**Figure 3:**
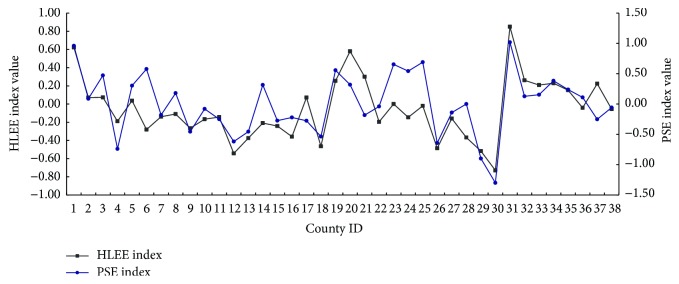
The curve of human living environmental evaluation (HLEE) index and public services evaluation (PSE) index (county ID is the same as it in Figure 2).

In Shenyang-Dalian urban agglomeration, Figure 3 presents good correlation between curve of public services evaluation (PSE) index and HLEE index (see Figure 3). The PSE index in Shenyang and Dalian Cities is higher than other counties, and it has big differences in these counties, which reflects the uneven development in the postal, medical, transport, and insurance. The abnormal data of PSE is found in industrial energy cities: Anshan, Benxi, and Tieling. Traditional energy economy can bring the convenience of the public service, but more environmental pollution and destruction may be caused by the traditional development of energy industry.

**Figure 4:**
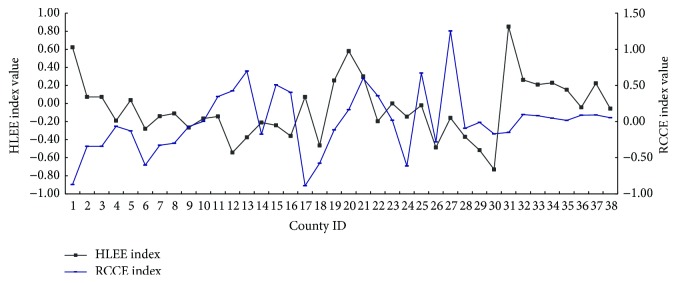
The curve of human living environmental evaluation (HLEE) index and resource carrying capacity (RCCE) index (county ID is the same as it in Figure 2).

It has a great difference between human living environmental evaluation (HLEE) index and resource carrying capacity (RCCE) index. When some counties have the small PSE index value, it can reflect that the pressure of environmental resources is small, such as Shenyang, Kangping, Yingkou, and Tieling (see Figure 4). When the HLEE indexes of these counties are higher, on the contrary, RCCE indexes are at the lower level, which show that the developing industrial cities have heavier load of resources carrying capacity.

**Figure 5:**
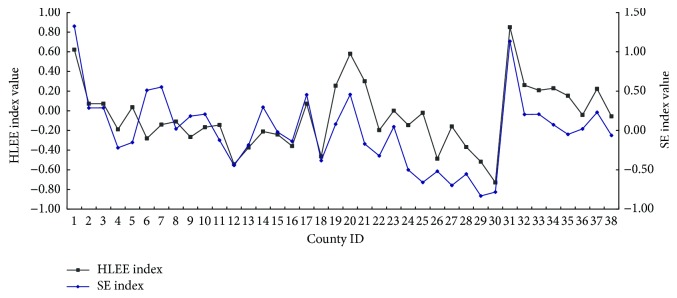
The curve of human living environmental evaluation (HLEE) index and socioeconomic evaluation (SE) index (county ID is the same as it in Figure 2).

The socioeconomic evaluation (SE) indexes of Shenyang and Dalian are higher than other counties, and the SE indexes of Fuxin County and Zhangwu County are lowest in the study area, where they have relatively backward economic development in Shenyang-Dalian urban agglomeration. Meanwhile, the HLEE indexes of these counties are lower than other counties. In economically developed regions, HLEE index increases with the increase of SE index (see Figure 5), including Shenyang, Liaoyang, Dalian, and Dandong.

### 4.4. Analysis of the Map of Evaluation Index

**Figure 6:**
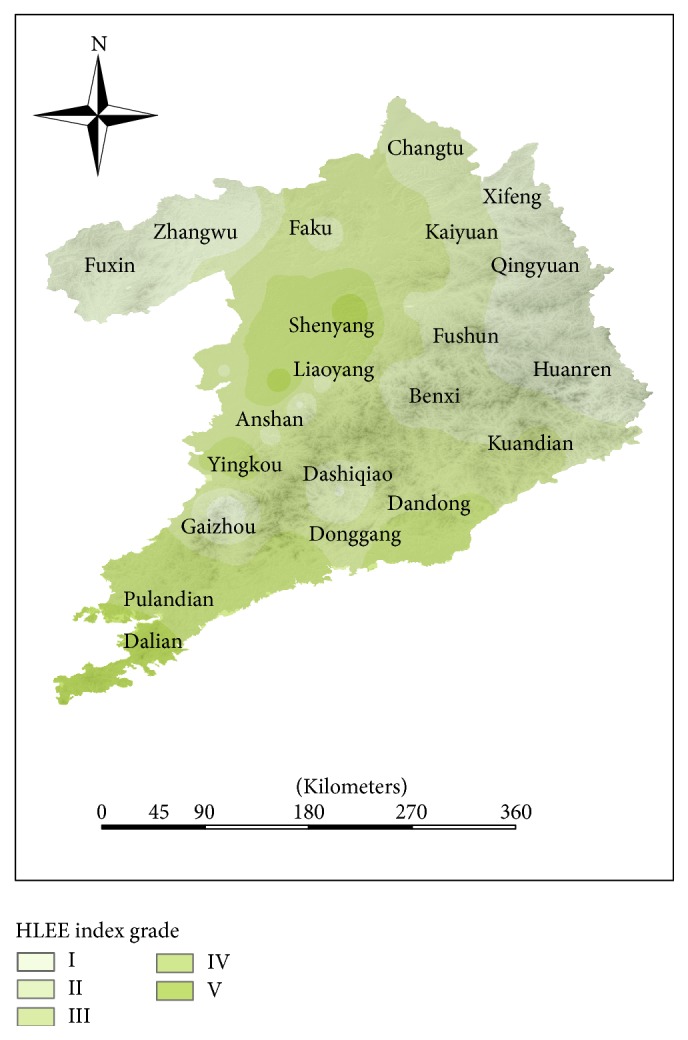
The distribution of human living environmental evaluation (HLEE) index in Shenyang-Dalian urban agglomeration.

The human living environmental evaluation index of Shenyang-Dalian urban agglomeration has been shown as in Figure 6. The HLEE indexes of central cities (Dalian and Shenyang) are higher than other counties, and the HLEE index of surrounding counties of these two central cities was better than the remote inland regions.

#### 4.4.1. Analysis of Resources Carrying Capacity Evaluation Index

**Figure 7:**
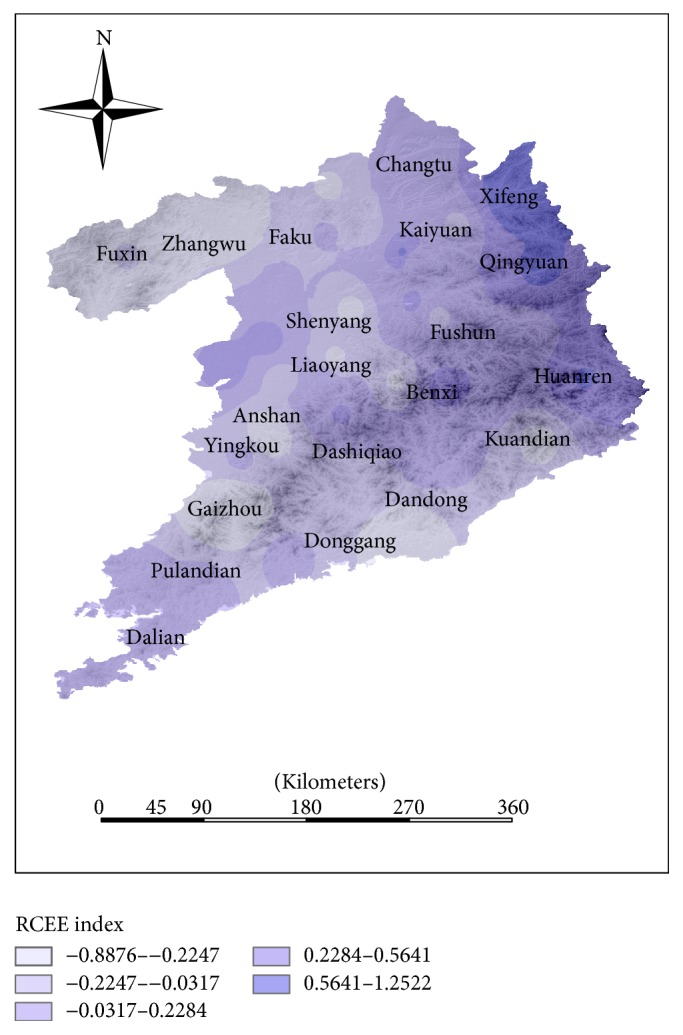
The distribution of resources carrying capacity evaluation (RCCE) index in Shenyang-Dalian urban agglomeration.

The high resources carrying capacity evaluation index is mainly distributed in the northeast region of Shenyang-Dalian urban agglomeration (see Figure 7), and its distribution is related to the distribution of energy consumption. It reflects that the regional economic growth is dependent on traditional energy consumed in this region. Due to the limit of technological innovation, development of Shenyang-Dalian urban agglomeration has the problem of product structure and extensive growth modes, which lead to the excessive use of resources. It mainly occurs in inland cities, for example, Xifeng, Qingyuan, and Huanren. Because of the small population growth and population density, the county has a lower RCCE index than other counties in this urban agglomeration.

#### 4.4.2. Analysis of Natural Environmental Evaluation Index

**Figure 8:**
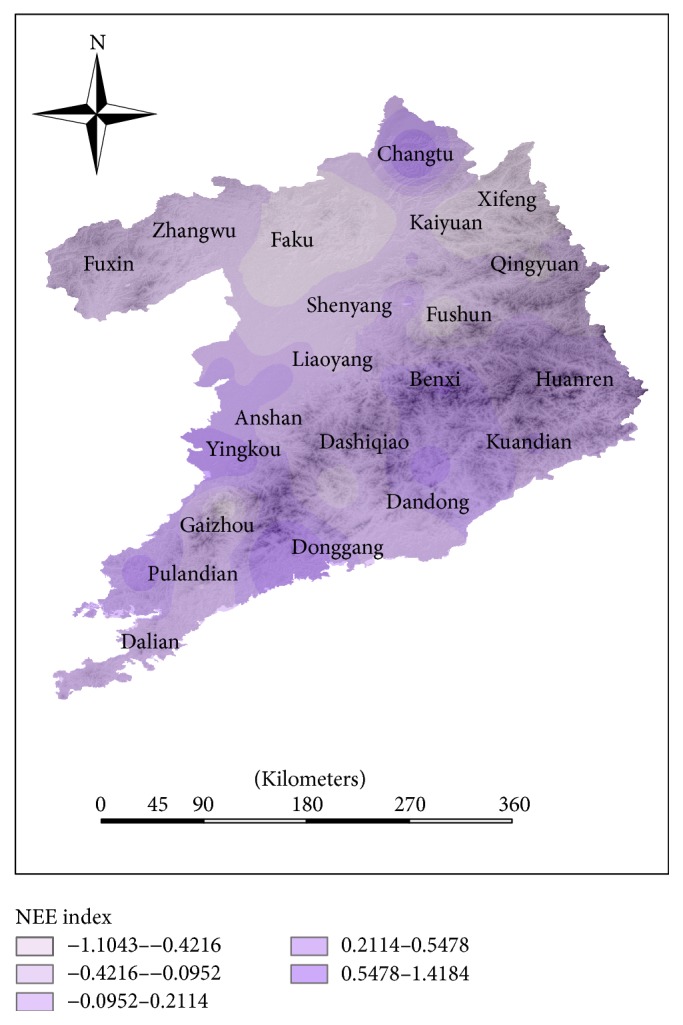
The distribution of natural environmental evaluation (NEE) index in Shenyang-Dalian urban agglomeration.

The natural environmental evaluation (NEE) indexes of some counties which are mainly distributed in the coastal counties and southern urban agglomeration are higher than the NEE index in inland counties (see Figure 8). The index system of the natural environment only includes climate factors and vegetation coverage factors. As shown in Figures 10 and 11, the distribution of climate evaluation index is similar to the distribution of NEE index and the distribution of vegetation coverage is different from the distribution of NEE index. It shows that climate is the dominant factor in the region's natural factors. In this region, Huanren and Kuandian are rich in natural resources. The northwest regions, where NEE index is relatively low, such as Fuxin and Faku, have large temperature difference and less vegetation cover. Coupled with the backwardness of economic development, the HLEE indexes in these counties are much lower than other counties.

#### 4.4.3. Analysis of Socioeconomic Evaluation Index

**Figure 9:**
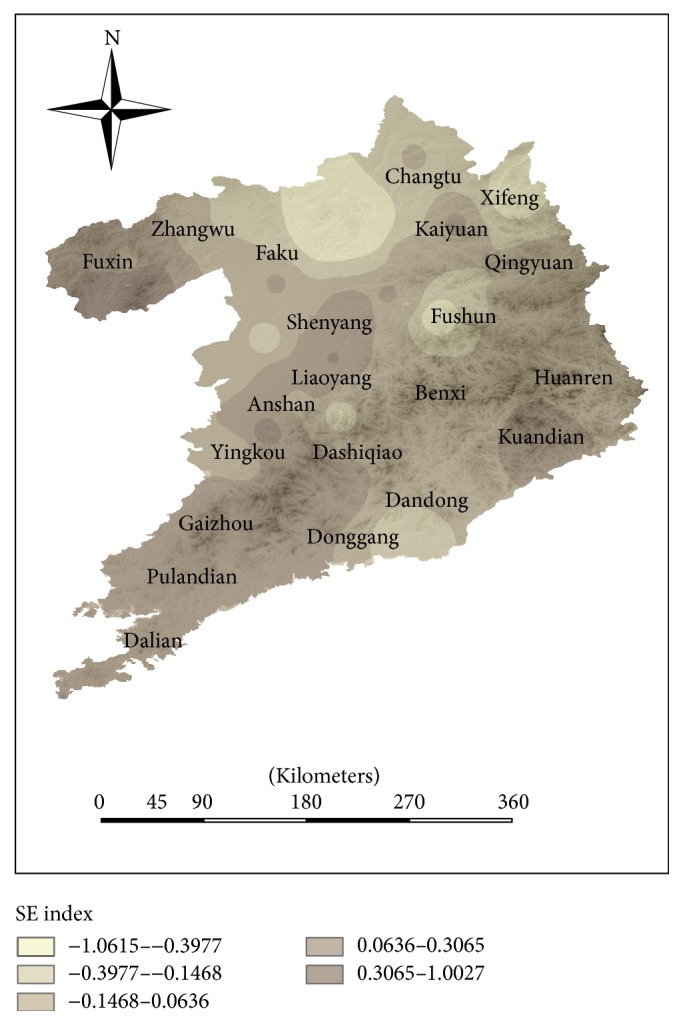
The distribution of socioeconomic evaluation (SE) index in Shenyang-Dalian urban agglomeration.

**Figure 10:**
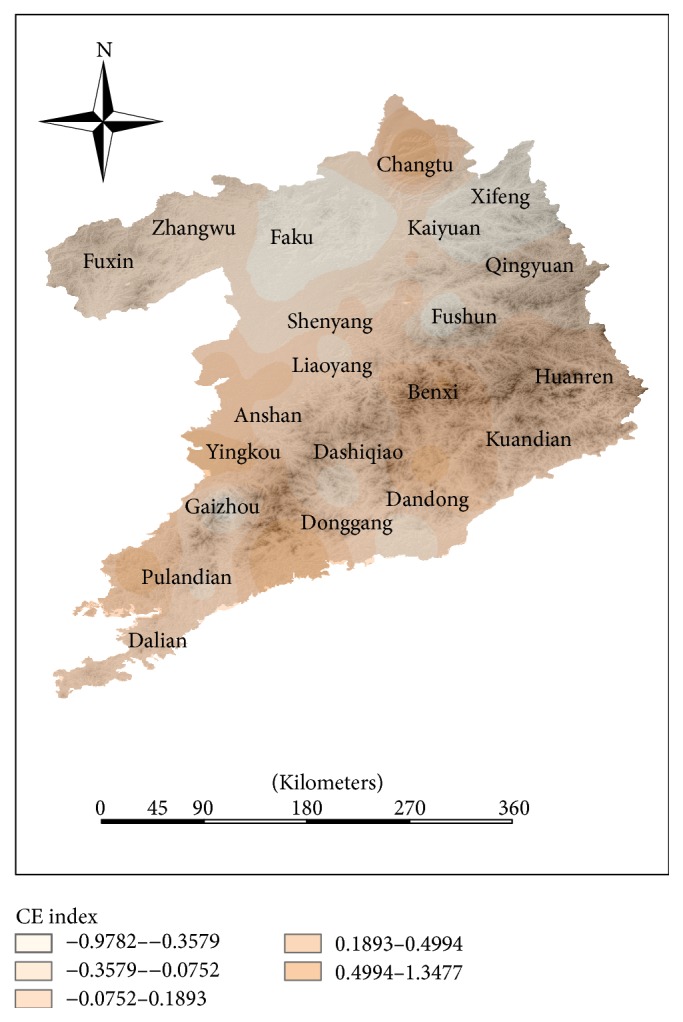
The distribution of climate evaluation (CE) index in Shenyang-Dalian urban agglomeration.

**Figure 11:**
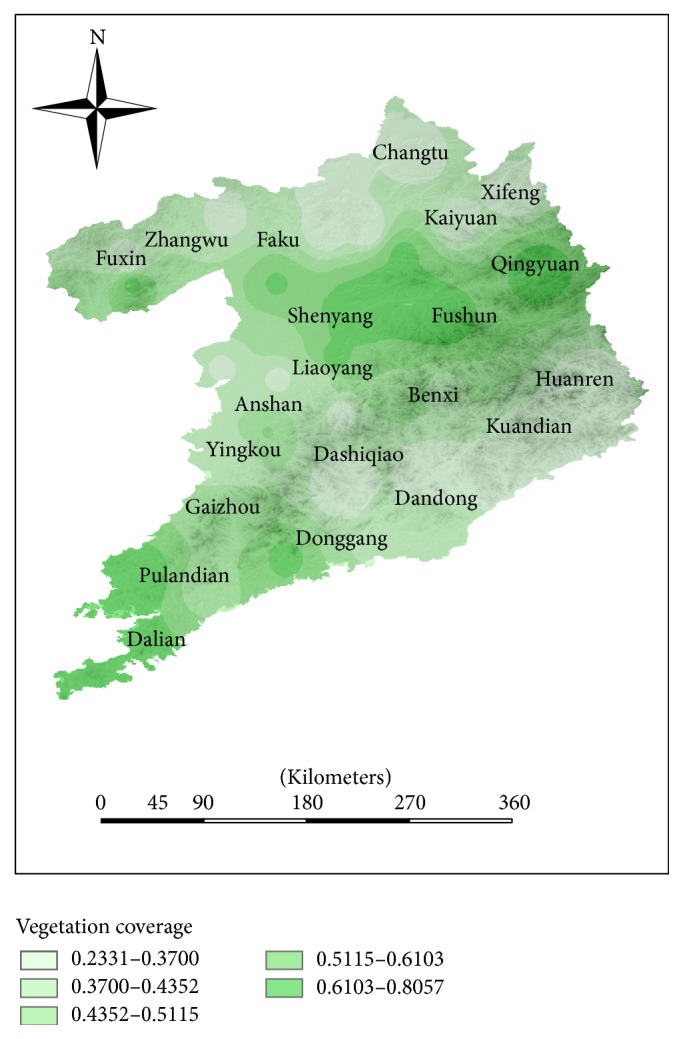
The distribution of vegetation coverage in Shenyang-Dalian urban agglomeration.

The distribution of the higher socioeconomic evaluation index is mainly in urban areas, such as Dalian, Shenyang, and other economic developed counties. This distribution is similar to distribution of economic development (see Figure 9). The development of economy, transportation, and municipal construction in Shenyang and Dalian is highest in this urban agglomeration, which leads to the higher HLEE index. Because economic structure is not reasonable, the economic growth of some counties, such as Kaiyuan, Faku, and Fushun, is less than the previous areas.

#### 4.4.4. Difference Analysis

Shenyang, Dalian, and Dandong are ranked in the forefront in the aspects of economic development and transport, which also has the higher HLEE index. Although the HLEE index in Anshan is relatively high, it is an energy industrial city, and it will be under enormous pressure between economic development and environmental protection. Because its population base is not consistent with the city size, the economic development of Tieling and Fushun can affect the development of other aspects, which means that the HLEE index is relatively low. Fushun and Tieling are the old industrial base and now are still the resource-consuming cities. Overall, the Shenyang-Dalian urban agglomeration has the advantage of energy and geographic position; however, the development in quality of human living environment is not coordinated.

### 4.5. The Planning Recommendations of Human Living Environment

There is a big difference in HLEE index in Shenyang-Dalian urban agglomeration. In the industrial energy consumption, counties have lower evaluation scores. Because of pursuing the economic development, some regions also do not transfer the extensive style of economic development to intensive development. Li et al. (2011) analyzed the human living environmental evaluation index in Chongqing and found that the human activities are the main reason to affect the HLEE index [7]. Their results are similar to this research. And this study suggests that economic development is the main factor affecting the quality of human living environment. In this region, economic development often cannot avoid the destruction of the environment. In order to improve the living environment in cities and counties, more conducive measures should be made in rational planning and development of urban agglomerations. First, the local government should pay attention to the construction of the ecological environment and scientific urban planning and construction. Second, protection of wetlands and afforestation can increase the green space and improve the ecological carrying capacity of resources. Third, development of the circular and intensive economy is the key measure to improve the human living environmental quality.

## 5. Conclusion

Combining PCA method with GIS software, this study developed the human living environmental evaluation model and quantitatively analyzed the human living environmental evaluation in a typical zone in Shenyang-Dalian urban agglomeration.

Based on this model, the human living environmental evaluation indexes of Dalian, Shenyang, and Liaoyang are higher than other counties. Among these counties, the human living environmental evaluation indexes of coastal counties are significantly higher than inland counties. Most study areas show that the range of the human living environmental evaluation indexes was at III, IV, and V levels, accounting for 80.01%. Based on these results, it could illustrate the regional human living environmental evaluation is in good condition.

Because of using the multiple factors model for assessing the regional human living environmental evaluation, the results closely reflect the real situation of Shenyang-Dalian urban agglomeration. As the evaluation factors are based on counties, it is difficult to know the counties' internal spatial pattern and subtle differences of human living environmental evaluation index. So evaluation accuracy can be enhanced by increasing more detailed information on the use of samples below the county level.

## Figures and Tables

**Table 1:** The human living environmental evaluation (HLEE) index system.

Resource carrying capacity evaluation (RCCE) index (*B*_1_)	
Population (*C*_1_)	(i) Population density (*D*_1_)
	(ii) The natural people growth rate (*D*_2_)
Land resource (*C*_2_)	(i) Per-capita wetland area (*D*_3_)
	(ii) Per-capita farmland area (*D*_4_)
Water resource (*C*_3_)	Per-capita water area (*D*_5_)
Energy (*C*_4_)	(i) Unit energy consumption (*D*_6_)
	(ii) Energy consumption per unit of GDP (*D*_7_)

Natural environmental evaluation (NEE) index (*B*_2_)	
Climate evaluation (CE) index (*C*_5_)	(i) Annual sunshine time (*D*_8_)
	(ii) Annual precipitation (*D*_9_)
	(iii) Relative humidity (*D*_10_)
	(iv) The annual average temperature (*D*_11_)
Ecological (*C*_6_)	Vegetation coverage (*D*_12_)

Socioeconomic evaluation (SE) index (*B*_3_)	
Public service evaluation (PSE) index (*C*_7_)	(i) The number of cultural and artistic venues (*D*_13_)
	(ii) Postal traffic per capita (*D*_14_)
	(iii) The number of doctors/ten thousand individuals (*D*_15_)
	(iv) Road density (*D*_16_)
	(v) Per capita library collection (*D*_17_)
	(vi) Urban medical insurance coverage (*D*_18_)
	(vii) Passenger turnover (*D*_19_)
Economy (*C*_8_)	(i) Rate/income ratio (*D*_20_)
	(ii) GDP growth (*D*_21_)
	(iii) GDP per capita (*D*_22_)
	(iv) Household consumption (*D*_23_)
	(v) The per capita disposable income (*D*_24_)
	(vi) Per-capita retail sales (*D*_25_)
Life and living (*C*_9_)	(i) Per-capita housing area (*D*_26_)
	(ii) Internet households rate (*D*_27_)
	(iii) Urban population density (*D*_28_)
	(iv) Family entertainment, education, and cultural services spending (*D*_29_)
Environment (*C*_10_)	(i) Drinking water quality compliance rate (*D*_30_)
	(ii) Sewage harmlessness (*D*_31_)

**Table 2:** Human living environmental evaluation grade in Shenyang-Dalian urban agglomeration.

Evaluation level	HLEEI value	Feature description
I	−0.72~−0.26	Unsuitable living environment, bad quality of life, and bad natural environment and public facilities

II	−0.26~−0.08	Less suitable living environment, low quality of life, and relatively bad natural environment and public facilities

III	−0.08~0.05	Suitable living environment, a little high quality of life, and relatively general natural environment and general public facilities

IV	0.05~0.15	Moderate suitable living environment, relatively high quality of life, relatively good natural environment, and relatively good public facilities

V	0.15~0.62	High suitable living environment, high quality of life, good natural environment, and good public facilities

**Table 3:** The results of principal component analysis (PCA).

Principal component	Initial eigenvalues		
	Eigenvalue (ei)	Contribution ratios (%)	Cumulative contribution (%)
1	5.185	23.567	23.567
2	3.999	18.178	41.745
3	1.994	9.063	50.808
4	1.824	8.292	59.100
5	1.485	6.752	65.852
6	1.461	6.639	72.491
7	1.003	4.557	77.048

**Table 4:** The grade proportion of human living environmental evaluation index.

Grade	Number of grid	Area (km^2^)	Area percentage (%)
I	3338	5590.44	5.34
II	9159	15337.06	14.65
III	17822	29857.54	28.52
IV	18741	31396.48	29.99
V	13440	22508.32	21.50

## References

[1] Doxiadis C. A. (1968). *Ekistics: An Introduction to the Science of Human Settlements*.

[2] Gilbert A. (1998). An urbanizing world: global report on human settlements. *Habitat International*.

[3] Henderson F. M., Xia Z.-G. (1997). SAR applications in human settlement detection, population estimation and urban land use pattern analysis: a status report. *IEEE Transactions on Geoscience and Remote Sensing*.

[4] Jenerette G. D., Harlan S. L., Brazel A., Jones N., Larsen L., Stefanov W. L. (2007). Regional relationships between surface temperature, vegetation, and human settlement in a rapidly urbanizing ecosystem. *Landscape Ecology*.

[5] Pickett S. T. A., Burch W. R., Dalton S. E., Foresman T. W., Grove J. M., Rowntree R. (1997). A conceptual framework for the study of human ecosystems in urban areas. *Urban Ecosystems*.

[6] Luck M. A., Jenerette G. D., Wu J., Grimm N. B. (2001). The urban funnel model and the spatially heterogeneous ecological footprint. *Ecosystems*.

[7] Li Y., Liu C., Zhang H., Gao X. (2011). Evaluation on the human settlements environment suitability in the Three Gorges Reservoir Area of Chongqing based on RS and GIS. *Journal of Geographical Sciences*.

[8] Alberti M., Marzluff J. M., Shulenberger E., Bradley G., Ryan C., Zumbrunnen C. (2003). Integrating humans into ecology: opportunities and challenges for studying urban ecosystems. *BioScience*.

[9] Emmanuel R. (2005). Thermal comfort implications of urbanization in a warm-humid city: the Colombo Metropolitan Region (CMR), Sri Lanka. *Building and Environment*.

[10] Wilson J. S., Clay M., Martin E., Stuckey D., Vedder-Risch K. (2003). Evaluating environmental influences of zoning in urban ecosystems with remote sensing. *Remote Sensing of Environment*.

[11] Parker D. C., Manson S. M., Janssen M. A., Hoffmann M. J., Deadman P. (2003). Multi-agent systems for the simulation of land-use and land-cover change: a review. *Annals of the Association of American Geographers*.

[12] Plummer S. E. (2000). Perspectives on combining ecological process models and remotely sensed data. *Ecological Modelling*.

[13] Zhang Q., Zhu C., Liu C. L., Jiang T. (2005). Environmental change and its impacts on human settlement in the Yangtze Delta, P.R. China. *Catena*.

[14] Peter N., Joe F., Mike B. (1998). *Environmental Indicators for National State of the Environment Reporting: Human Settlements*.

[15] Pickett S. T. A., Cadenasso M. L., Grove J. M. (2001). Urban ecological systems: linking terrestrial ecological, physical, and socioeconomic components of metropolitan areas. *Annual Review of Ecology and Systematics*.

[16] Munda G., Nijkamp P., Rietveld P. (1994). Qualitative multicriteria evaluation for environmental management. *Ecological Economics*.

[17] Mertens B., Lambin E. F. (1997). Spatial modelling of deforestation in southern Cameroon: spatial disaggregation of diverse deforestation processes. *Applied Geography*.

[18] Shaw G., Wheeler D. (1985). *Statistical Techniques in Geographical Analysis*.

[19] Apan A. A. (1997). Land cover mapping for tropical forest rehabilitation planning using remotely-sensed data. *International Journal of Remote Sensing*.

[20] Li A., Wang A., Liang S., Zhou W. (2006). Eco-environmental vulnerability evaluation in mountainous region using remote sensing and GIS—a case study in the upper reaches of Minjiang River, China. *Ecological Modelling*.

